# The Role of Angiotensin Receptor Blockers in the Personalized Management of Diabetic Neuropathy

**DOI:** 10.3390/jpm12081253

**Published:** 2022-07-30

**Authors:** Danai-Thomais Kostourou, Dimitrios Milonas, Georgios Polychronopoulos, Areti Sofogianni, Konstantinos Tziomalos

**Affiliations:** First Propedeutic Department of Internal Medicine, Medical School, Aristotle University of Thessaloniki, AHEPA Hospital, Thessaloniki 54636, Greece; danaekost@yahoo.gr (D.-T.K.); milonasdim.ver@gmail.com (D.M.); giourkaspol@gmail.com (G.P.); aretisofo@hotmail.com (A.S.)

**Keywords:** diabetes mellitus, diabetic neuropathy, angiotensin receptor blockers

## Abstract

Neuropathy is a frequent complication of diabetes mellitus (DM) and is associated with the increased risk ofamputation and vascular events. Tight glycemic control is an important component inthe prevention of diabetic neuropathy. However, accumulating data suggest that angiotensin receptor blockers (ARBs) might also be useful in this setting. We discuss the findings of both experimental and clinical studies that evaluated the effects of ARBs on indices of diabetic neuropathy. We also review the implicated mechanisms of the neuroprotective actions of these agents. Overall, it appears that ARBs might be a helpful tool for preventing and delaying the progression of diabetic neuropathy, but more data are needed to clarify their role in the management of this overlooked complication of DM.

## 1. Introduction

Type 2 diabetes mellitus (T2DM) is becoming increasingly prevalent and currently affects approximately 14.3% of the adult US population [1]. Moreover, a further 38.0% of the adult US population has prediabetes, i.e., impaired fasting glucose or impaired glucose tolerance [1]. Diabetic neuropathy is a frequent complication of both T2DM and type 1 diabetes mellitus (T1DM), and it is present in approximately 3.5–9.4% of patients who havehad T1DM for 1–5 years and in 28% of patients who havehad T2DM for > 4 –7 years [2,3,4]. The prevalence of diabetic neuropathy increases with age and diabetes duration, and it is higher in patients with poor glycemic control [5,6,7,8,9,10]. Diabetic neuropathy can affect almost all parts of the nervous system, and its most common forms are chronic distal symmetric sensorimotor polyneuropathy and autonomic neuropathy [7]. The former increases the risk for foot ulcer, amputation, and death [7,11,12,13]. On the other hand, cardiovascular autonomic neuropathy is present in approximately one-third of patients with T2DM and in a similar proportion of patients who have had T1DM for approximately 25 years [5,6]. More importantly, cardiovascular autonomic neuropathy is associated with increased vascular morbidity and mortality in patients with either T1DM or T2DM [7,14,15,16,17].

**Figure 1:**
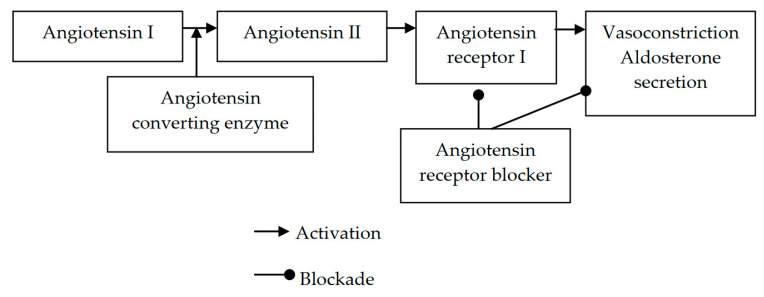
Mechanism of action of angiotensin receptor blockers.

Randomized controlled trials have showed that tight glycemic control prevents and delays the progression of both peripheral and autonomic neuropathy in T1DM [4,5,18,19,20]. In contrast, the effects of tight glycemic control on T2DM-associated neuropathy are more controversial, with some reports showing benefits [21,22,23] and others having no effect [24,25]. Several agents have been used in patients with diabetic neuropathy, including aldose reductase inhibitors, antioxidants, and protein kinase C inhibitors, but their benefit is unclear [7]. Angiotensin receptor blockers (ARBs) are the antihypertensive agents of choice for hypertensive patients with diabetes mellitus (along with angiotensin-converting enzyme inhibitors, ACE-I) [26]. ARBs act by inhibiting the binding of angiotensin II (which is produced by the cleavage of angiotensin I by the angiotensin-converting enzyme) to angiotensin receptor I, thereby promoting vasodilation and inhibiting aldosterone secretion [27] (Figure 1). Accumulating experimental data and some small clinical studies suggest that ARBs might also have a role in the management of diabetic neuropathy. We summarize these data and discuss the implicated mechanisms.

## 2. Search Strategy

The PubMed database was reviewed for papers using the terms “diabetes”, “neuropathy”, “angiotensin receptor blocker”, “losartan”, “valsartan”, “candesartan”, “olmesartan”, “telmisartan” and “azilsartan”. The references of pertinent articles were also hand-searched for relevant papers.

## 3. Preclinical Studies

In an early study ofstreptozotocin-diabetic rats, the ARB ZD 8731 was given 1 month after the induction of diabetes and for a duration of 1 month [28]. ZD 8731 ameliorated both motor and sensory nerve conduction velocity (NCV) [28]. An increase in nerve capillary density was observed, which might have contributed to the improvement in NCV [28]. In contrast, ZD 8731 had no effect on these parameters in non-diabetic rats [28]. The same group of investigators assessed the effects of another ARB, ZD 7155, in streptozotocin-diabetic rats [29]. ZD 7155 was given for 1 month, either immediately after the induction of diabetes or after 1 month [29]. The amelioration of motor and sensory NCV and an improvement in nerve regeneration after experimental damage were observed regardless of the timing of treatment initiation [29]. The investigators confirmed an ARB-induced increase in nerve capillary density and also observed an augmentation of endoneurial blood flow [29]. In another study, olmesartan improved nerve regeneration in diabetic rats [29]. There was an increase in the production of the ciliary neurotrophic factor (a nerve growth factor), which might have played a role in this neurotrophic effect [30]. Interestingly, in vitro and animal studies have also reported the neurotrophic effect of olmesartan on spinal motor neurons in non-diabetic animals [31].In an in vitro study ofPC12 cells, both losartan and telmisartan reduced oxidative stress, but only telmisartan prevented glucose-induced apoptosis [32]. In more recent studies, both losartan and telmisartan prevented the development of neuropathy in diabetic rat models [33,34]. The principal findings of major preclinical studies that evaluated the effect of ARBs on diabetic neuropathy are summarized in Table 1.

## 4. Clinical Studies

In contrast to these promising experimental data, early clinical studies that assessed the effects of ARBs on diabetic neuropathy yielded negative results [35,36]. In normotensive patients with T2DM and microalbuminuria, treatment with losartan for 12 weeks did not improve peripheral or autonomic neuropathy [35]. In another early study onpatients with T1DM or T2DM, treatment with losartan for 12 months did not improve cardiovascular autonomic function or the vibration-perception threshold [36]. However, in another study, treatment with losartan for 12 months improved autonomic nervous function in normotensive patients with autonomic neuropathy due to either T1DM or T2DM [37]. Treatment with quinapril also ameliorated autonomic neuropathy, in accordance with previous findings [38,39]. Interestingly, the losartan/quinapril combination appeared to be more beneficial than either monotherapy [37]. However, the vibration-perception threshold did not change with either losartan or quinapril [37]. These findings, although preliminary, suggest that the beneficial effectsof ARB on diabetic neuropathy become apparent only after long-term treatment, and that autonomic neuropathy improves more with these agents than peripheral neuropathy [37]. However, a study onhypertensive patients with diabetic nephropathy showed an improvement in the low-tohigh-frequency ratio (an index of sympathovagal balance) after only 12 weeks of treatment with losartan or telmisartan [40]. The principal findings of major clinical studies that evaluated the effect of ARBs on diabetic neuropathy are summarized in Table 2.

## 5. Studies Onnon-Diabetic Patients

Studies onnon-diabetic patients have also suggested an improvement in various indices of cardiovascular autonomic neuropathy with ARB treatment [41]. Several different ARBs improved cardiac baroreceptor sensitivity in different populations, including patients with hypertension [42]. ARBs werealso observed to increase heart rate variability (HRV), another index of cardiovascular autonomic function, in obese prehypertensive patients [43] and in patients with hypertension [44], ischemic cardiomyopathy [45], or idiopathic dilated cardiomyopathy [46]. ARBs also decreased the low-tohigh-frequency ratio in hypertensive patients [44]. However, other reports, mostly in hypertensive patients, did not show any benefit of ARBs with regard tothese parameters [45,46,47,48,49,50,51,52,53]. In addition, some studies reported an improvement in some parameters of cardiovascular autonomic function (i.e., baroreflex sensitivity), but not in others (i.e., HRV) [54]. Regarding the effects of ARBs on cardiovascular autonomic neuropathy as compared with other antihypertensive agents, the existing evidence is also controversial. Compared with beta-blockers in hypertensive patients, atenolol increased HRV and baroreflex sensitivity, whereas irbesartan and fosinopril had no effect [55]. In another study onhypertensive patients, losartan improved baroreflex sensitivity and did not affect HRV, whereas atenolol did not change baroreflex sensitivity and reduced HRV despite a similar BP reduction [56]. Several studies compared the effects of ARBs and ACE-Is on cardiovascular autonomic function, and most reported comparable improvements [57,58,59]. The combination of the two classes yielded greater reductions than monotherapy ineither class [57]. However, in one report onpatients with heart failure, lisinopril increased HRV, whereas valsartan had no effect [60]. Neither valsartan nor lisinopril had any effect on baroreflex sensitivity [60]. A recent observational study also suggested that treatment with either ARBs or ACE-Is protects against platinum-induced sensory neuropathy [61]. These discrepant results are partly due to the small number of patients and differences in patients’ characteristics, duration of treatment, and index of autonomic function. It is also unclear whether these findings in non-diabetic subjects can be extrapolated to diabetic patients. The principal findings of major clinical studies that evaluated the effect of ARBs on neuropathy in non-diabetic patients are summarized in Table 3.

## 6. ARBs and Erectile Dysfunction

Diabetic neuropathy might also manifest as erectile dysfunction, which is present in approximately 60% of patients who have had T1DM or T2DM for > 10–20 years [62,63]. Studies onstreptozotocin-diabetic rats and aged rats showed an improvement in erectile function with ARB treatment [64,65,66]. ARBs also ameliorated erectile dysfunction in hypertensive patients with [67] or without the metabolic syndrome, which is a prediabetic condition [68,69]. However, it has not been evaluated whether ARBs improve erectile function in diabetic patients.The principal findings of major clinical studies that evaluated the effect of ARBs on erectile dysfunction are summarized in Table 4.

## 7. Putative Mechanisms of the Effects of ARBs on Diabetic Neuropathy

**Figure 2:**
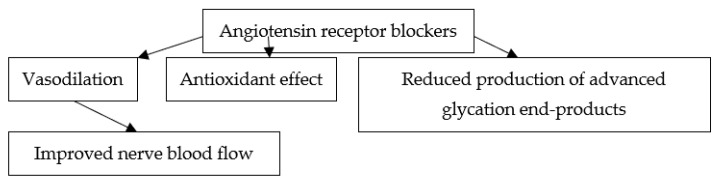
Major mechanisms potentially implicated in the beneficial effects of angiotensin receptor blockers on diabetic neuropathy.

Several mechanisms appear to account for the beneficial effects of ARBs on diabetic neuropathy. ARBs might confer neuroprotection by improving nerve blood flow through their vasodilating properties [70]. Interestingly, in animal models of diabetic neuropathy, angiotensin II reduced endoneurial blood flow more than in non-diabetic animals [71]. Oxidative stress and advanced glycation end-products also appear to play a role in the pathogenesis of diabetic neuropathy [72,73]. On the other hand, ARBs exert antioxidant effects [74,75] and reduce the production of advanced glycation end-products [76]. Accordingly, it was suggested that these actions might play a role in the neuroprotective effects of ARBs [77]. Several prospective studies showed that hypertension increases the risk ofneuropathy in diabetic patients [8,9]. However, in several randomized controlled trials involving patients with T2DM, more aggressive antihypertensive treatment did not prevent or delay the progression of neuropathy as compared with less tight blood pressure control [78,79,80,81]. Nevertheless, beta-blockers, calcium channel blockers, and ACE-Is were used in the latter trials, and it remains to be established whether ARBs can improve diabetic neuropathy independently of their blood pressure-lowering effects. Regarding erectile function, the beneficial effects of ARBs are partly due to the inhibition of angiotensin II, which is locally produced in the corpus cavernosum and directly terminates erection [82,83,84]. ARBs also improved erectile function through the suppression of oxidative stress and an increase in the expression of endothelial nitric oxide synthase in the corpus cavernosum [64]. Whether the improvement in diabetic neuropathy plays a role in the amelioration of erectile dysfunction during ARB treatment remains to be established.The major mechanisms potentially implicated in the beneficial effects of ARBs on diabetic neuropathy are shown in Figure 2.

## 8. Indications for ARB Therapy

AllARBs are indicated for the management of hypertension. Losartan and irbesartan are also indicated for the management of chronic kidney disease in hypertensive patients with T2DM. Losartan, valsartan, and candesartan are also indicated for the management of patients with heart failure and reduced ejection fraction (i.e.,≤ 40%) if ACE-Is are contraindicated or not tolerated. Valsartan is also indicated for the treatment ofpatients with a recent myocardial infarction and symptomatic heart failure or asymptomatic left ventricular systolic dysfunction. Losartan is also indicated for the prevention of ischemic stroke in hypertensive patients with left ventricular hypertrophy diagnosed viaelectrocardiogram. Telmisartan is also indicated for the prevention of cardiovascular events in patients with established cardiovascular disease and in patients with T2DM and target organ damage.

## 9. Conclusions

Emerging experimental and clinical data suggest than ARBs might be a useful tool for preventing and delaying the progression of diabetic neuropathy. It is also possible that the combination of ARBs with other interventions that improve diabetic neuropathy (mainly tight glycemic control) might yield an incremental benefit [85]. Indeed, in the Steno-2 study, intensified multifactorial intervention that included an ACE-I, an ARB, or both, but also lifestyle changes, intensive glucose- and lipid-lowering treatment, and aspirin delayed the progression of autonomic neuropathy in patients with T2DM and microalbuminuria [85]. However, peripheral neuropathy was not affected [85]. Clearly, more data are needed to clarify the potential role of ARBs in the management of this overlooked complication of diabetes.

## Figures and Tables

**Table 1:** Principal findings of preclinical studies that evaluated the effect of angiotensin receptor blockers (ARBs) on diabetic neuropathy.

Ref.	ARB	Animal Model	Major Findings
[28]	ZD 8731	Streptozotocin-diabetic rats	ZD 8731 ameliorated motor and sensory nerve conduction velocity and increased nerve capillary density
[29]	ZD 7155	Streptozotocin-diabetic rats	ZD 7155 ameliorated motor and sensory nerve conduction velocity, improved nerve regeneration, and increased nerve capillary density and endoneurial blood flow
[30]	Olmesartan	Diabetic rats	Olmesartan improved nerve regeneration and increased the production of the ciliary neurotrophic factor

**Table 2:** Principal findings of major clinical studies that evaluated the effect of angiotensin receptor blockers (ARBs) on diabetic neuropathy.

Ref.	*n*	Study Population	ARB	Treatment Duration (Months)	Major Findings
[35]	25	Normotensive patients with T2DM and microalbuminuria	Losartan	1	Losartan had no effect on peripheral or autonomic neuropathy
[36]	44	Patients with T1DM or T2DM	Losartan	12	Losartan had no effect on cardiovascular autonomic function or vibration-perception threshold
[37]	62	Normotensive patients with T1DM or T2DM and autonomic neuropathy	Losartan	12	Losartan improved autonomic nervous function but did not affect vibration-perception threshold

T2DM: type 2 diabetes mellitus; T1DM: type 1 diabetes mellitus.

**Table 3:** Principal findings of major clinical studies that evaluated the effect of angiotensin receptor blockers (ARBs) on neuropathy in non-diabetic patients.

Ref.	*n*	Study Population	ARB	Treatment Duration (Months)	Major Findings
[43]	50	Prehypertensive obese patients	Losartan	4	Losartan decreased heart sympathetic activity
[47]	57	Hypertensive patients	Telmisartan	2	Telmisartan increased heart parasympathetic activity
[49]	25	Young males	Eprosartan	7 days	Eprosartan lowered heart rate variability and baroreflex gain

**Table 4:** Principal findings of major clinical studies that evaluated the effect of ARBs on erectile dysfunction.

Ref.	*n*	Study Population	ARB	Treatment Duration (Months)	Major Findings
[67]	1069	Hypertensive males with metabolic syndrome	Irbesartan	6	Irbesartan improved erectile function, orgasmic function, and intercourse satisfaction
[68]	164	Hypertensive males	Losartan	3	Losartan improved sexual satisfaction and increased the frequency of sexual activity
[69]	3502	Hypertensive males	Valsartan	6	Valsartan improved erectile function, orgasmic function, and intercourse satisfaction; increased sexual desire
